# Synchronous Metastatic Clear Cell Renal Cell Carcinoma to the Gallbladder With Metachronous Contralateral Recurrence

**DOI:** 10.7759/cureus.15007

**Published:** 2021-05-13

**Authors:** Daniel P Pierce, Aaron S Dahmen, David J Hernandez

**Affiliations:** 1 Department of Urology, University of South Florida, Tampa, USA

**Keywords:** renal clear cell carcinoma, metastasis, gallbladder, cancer, recurrence, contralateral, metachronous, synchronous

## Abstract

Renal cell carcinoma (RCC) classically metastasizes to the lungs, bones, adrenals, lymph nodes, liver, and brain. RCC metastasis to the gallbladder is rare occurring in less than 1% of metastases. We present a case of a 60-year-old male who at initial diagnosis of his large left renal mass was incidentally found to have a gallbladder mass. He underwent simultaneous open radical nephrectomy and cholecystectomy with pathology confirming solitary metastatic clear cell RCC (ccRCC). The patient chose surveillance and was without evidence of disease for three years. At three years, imaging showed a 2 cm contralateral renal mass which was cryoablated percutaneously. This case demonstrates not only the importance of a thorough review of initial and surveillance imaging but also of maintaining a broad differential for other solid organ masses in the setting of a primary RCC of the kidney.

## Introduction

Renal cell carcinoma (RCC), more specifically the clear cell variant, accounts for up to 70% of all renal cell cancers. Not infrequently, patients are found to have distant metastasis often at the time of presentation or a subsequent recurrence years after an initial diagnosis and treatment. Most commonly, when clear cell RCC (ccRCC) metastasizes to a solid organ it is found in the lungs (6.19%), but it is also frequently identified in the liver, bone, and brain [1]. On the contrary, very rarely (0.58%) is ccRCC found in the gallbladder, which is usually discovered incidentally during autopsy [2]. We present a case of isolated metastatic ccRCC to the gallbladder in a patient with no prior oncologic history discovered during initial surgical resection of the primary tumor, who later developed a new renal mass on the contralateral kidney.

## Case presentation

**Figure 1:**
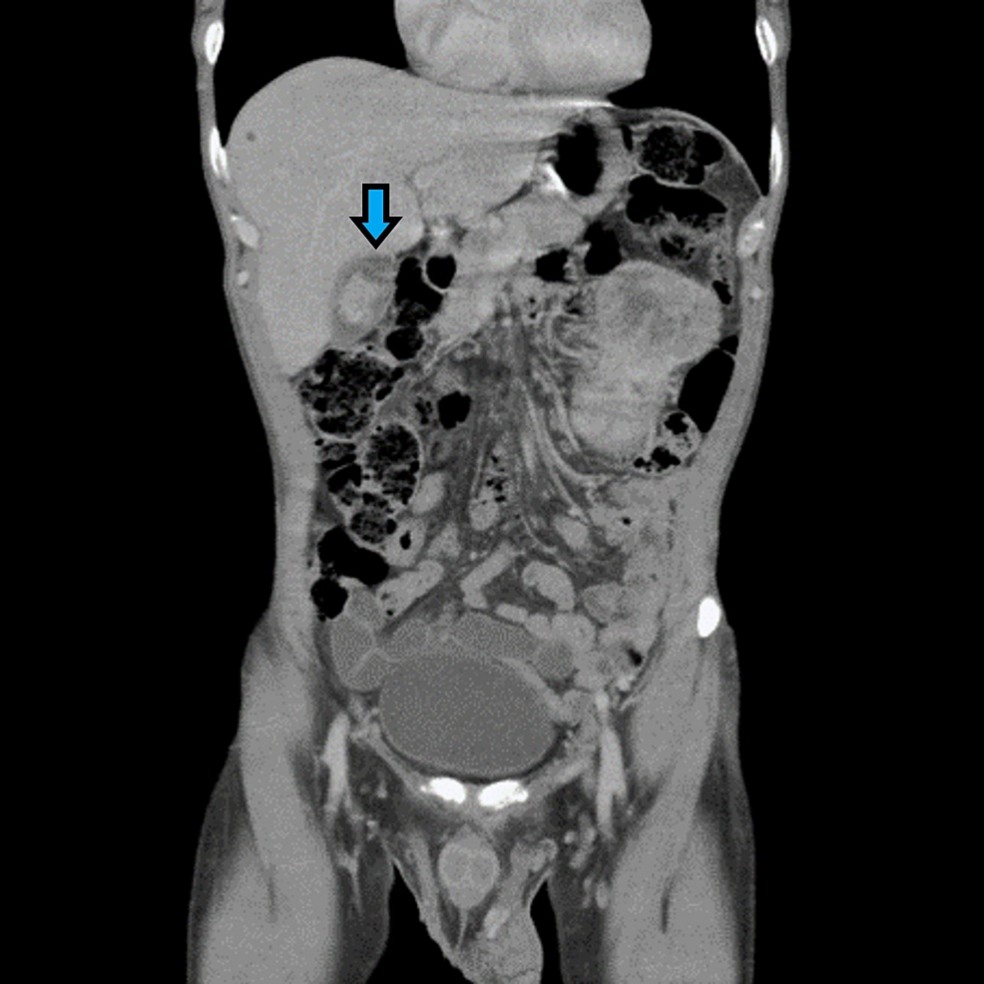
CT abdomen/pelvis with contrast demonstrating a soft tissue mass within the gallbladder in addition to a large left renal mass demonstrating both solid and complex cystic components.

A 60-year-old Egyptian male originally presented with syncope and acute -onset, sharp left flank, and left upper quadrant abdominal pain for four days associated with a three-hour loss of consciousness. The patient endorsed syncope upon rising from a chair at which point he slowly collapsed to the ground under the support of a nearby friend. There was no history of fever, hematuria, dysuria, frequency, urgency, or incomplete emptying. There was no association of chest pain or palpitations prior to the syncopal episode, and the patient had reported a few episodes of presyncope in the past associated with stress or sleep deprivation. Vitals upon admission were all within normal limits with a blood pressure of 126/80 mmHg, pulse 89 beats per minute, respiratory rate 22 breaths per minute, and temperature 98.1 °F. A review of systems was positive for a 20lb unintentional weight loss over several months. He was a lifetime nonsmoker with no personal history of cancer and only significant family history of a brother with prostate cancer. His original creatinine obtains on admission was 1.0 mg/dL. A non-contrast CT of the head was obtained while in the ED and was negative for any intracranial abnormality. A contrast-enhanced CT of the abdomen and pelvis was obtained and demonstrated a 5 mm left lower lobe pulmonary nodule, an irregular soft tissue density within the gallbladder suggestive of cholelithiasis vs a possible mass, and a large mass replacing most of the left kidney measuring 13.8 x 11.9 x 10.3 cm with scattered areas of calcification appearing both solid and cystic. No definite left renal vein extension and a grossly normal-appearing right kidney were appreciated as seen in Figure 1. An MRI of the abdomen was subsequently obtained which confirmed the absence of renal vein involvement.

Cardiology was initially consulted to evaluate for potential causes of the syncope and an echocardiogram was ordered in addition to placing the patient on telemetry. The echocardiogram revealed a normal-sized left and right ventricle with an estimated ejection fraction in the range of 55-60%. Additionally, the electrocardiogram (EKG) and telemetry strips demonstrated normal sinus rhythm suggesting the most likely cause of syncope to be orthostatic in nature and did not require a formal neurology consultation as there was no recurrence or enduring effects of this episode. At no point during his hospital stay did the patient experience any sustained and significant vital sign derangements. Four days after the original presentation and after a complete evaluation by urology, the patient was taken for an open left radical nephrectomy and cholecystectomy. Intraoperative findings demonstrated a large multilobulated left renal mass with extremely large serpiginous vessels extending down to the inguinal canal and up the left paracolic gutter. Frozen sections revealed negative surgical margins and a gallbladder positive for metastatic ccRCC. He was referred to medical oncology for stage IV, pT3aNxM1, with imaging negative for brain or bony metastasis. With negative margins and no evidence of disease, the patient elected for surveillance and did not pursue any additional adjuvant therapy. There were no additional significant findings at his initial postoperative visit. His postoperative creatinine remained relatively unchanged at 1.3 mg/dL. His follow-up was arranged on an as-needed basis with urology as he continued his primary surveillance with oncology.

**Figure 2:**
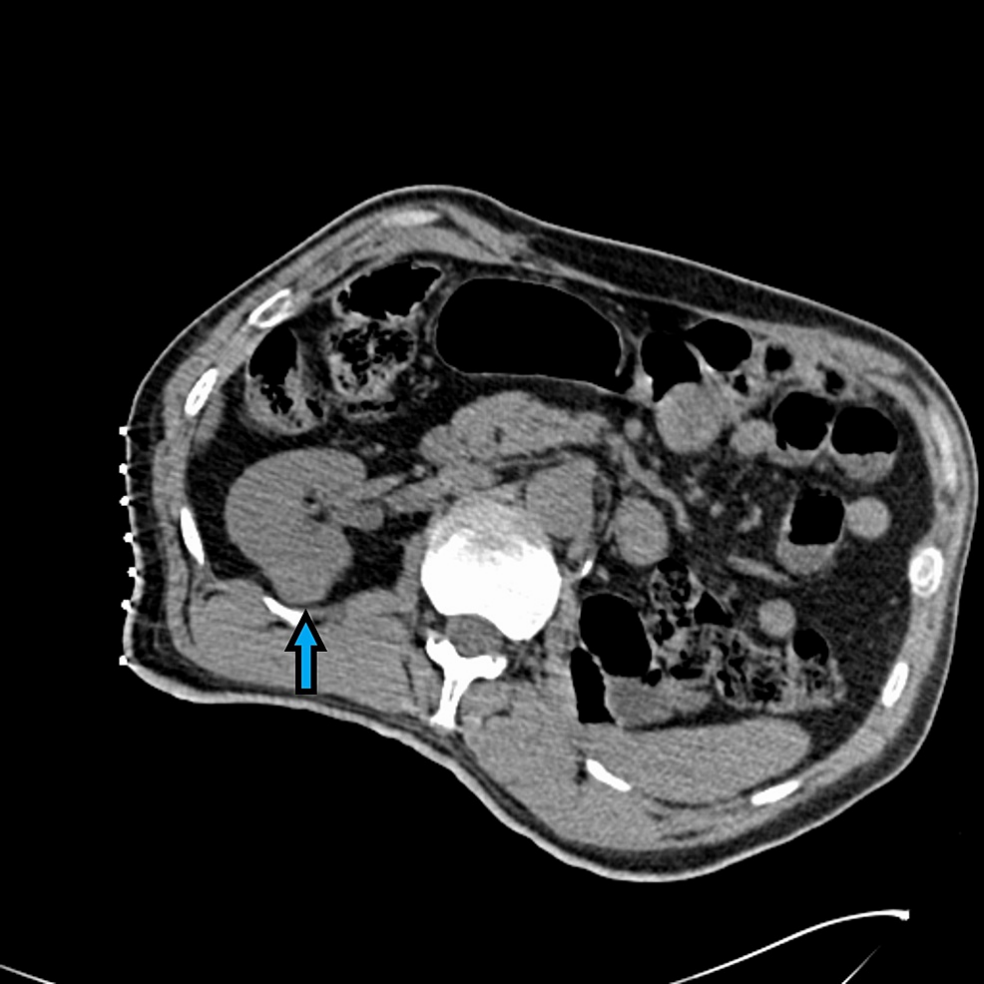
Renal CT demonstrating an exophytic soft tissue mass along the posterior border of the mid pole of the right kidney.

Subsequent surveillance imaging did not demonstrate any clinically significant change in his pulmonary nodule, nor did he demonstrate any recurrence of the disease. At almost three years postoperatively, the patient was noted to have a 2.5 x 1.7 cm exophytic right mid pole posterior renal mass as seen in Figure 2. The patient then underwent CT-guided right cryoablation of the renal mass three years from his original presentation. Since his ablation, he has been recurrence-free.

## Discussion

The most common sites of distant metastasis in terms of primary RCC are the lungs, bones, adrenals, lymph nodes, liver, and brain [3]. Of all patients diagnosed with primary RCC, approximately 1/3 of these will demonstrate metastasis at initial presentation, while 25-50% of patients will develop metastatic recurrence following treatment of the primary neoplasm. In a review of available literature, metastatic RCC to the gallbladder is extraordinarily rare, representing less than 1% of all metastatic RCC. The majority of these have been found in autopsies though still representing only 0.58% of cases with metastatic RCC [2]. We were able to identify 59 case reports presenting metastatic RCC to the gallbladder [4].

Other more common metastatic cancers to the gallbladder include melanoma, breast, colon, and pancreas. RCC is known for its ability of hematogenous spread, and some investigators have suggested this as a possible explanation for the connection of RCC and gallbladder metastasis [5]. On the contrary, it is worth noting that in the available literature reviews on cases of metastatic RCC to the gallbladder there appears to be no associated laterality or higher correlation of right-sided renal masses with gallbladder involvement. The biliary spread has also been suggested as a possible mechanism, but this has been thought to be less likely as there has not been sufficient evidence of tumor extension into the biliary ducts or liver and in fact all tumors reported have been present on the luminal rather than the serosal surface. Therefore, the current prevailing opinion is such that metastasis is truly a systemic disease rather than any direct involvement of the associated primary renal tumor [6].

In the review of the published cases, there are several notable aspects for clinicians to be aware of. In contrast to what we present here, most metastatic lesions to the gallbladder are metachronous [4]. Rarely they are identified symptomatically, but rather incidentally, on surveillance imaging, or frequently postmortem. Despite this, when identified symptomatically, they have presented with either symptoms of biliary colic or cholecystitis [7]. While imaging can suggest an underlying disease process within the gallbladder, whether primary or secondary, there are no discrete imaging findings in the literature to contrast a primary from the secondary process and are frequently identified as either involving gallbladder wall thickening or an intraluminal polypoid lesion.

Identification and resection of solitary metastatic lesions in addition to the primary has a known survival benefit, and in particular, successful outcomes have been published following resection of gallbladder metastases when R0 resections are performed [4, 8, 9]. While rare, identification of a gallbladder lesion in a patient with a history of RCC should prompt consideration of renal metastasis and subsequent cholecystectomy. As demonstrated in our patient, who presented with oligometastatic disease, he underwent radical nephrectomy and simultaneous cholecystectomy without evidence of subsequent local recurrence. Interestingly and unique to the literature, he presented three years following treatment with a contralateral renal lesion for which he underwent cryoablation and has done well since without further evidence of recurrence. It is unclear if this represented a recurrence/metastatic disease versus a second primary RCC. A few reports of metastasis exist years after resection of a renal cell primary tumor, which likely could be present in this case, unfortunately, no biopsy was obtained prior to cryoablation [10].

## Conclusions

This case presents a rare case of ccRCC metastatic to the gallbladder at the time of original diagnosis without evidence of metastatic disease elsewhere in the body. This patient developed either a recurrence or second primary tumor of the contralateral kidney years later but is otherwise now without evidence of disease. This case stresses the importance of thoroughly reviewing imaging in all cases, but especially for cancer staging. Metastatic ccRCC to the gallbladder remains a rare finding, but as more cases are reported there is likely benefit to further exploring trends as well as continuing to keep RCC within the working differential when the discovery of gallbladder lesions is made during staging workup for primary RCC.
